# Implementation of Plasma Fractionation in Biological Medicines Production

**DOI:** 10.15171/ijb.1401

**Published:** 2016-12

**Authors:** Kamran Mousavi Hosseini, Mehran Ghasemzadeh

**Affiliations:** Biotechnology Department, Blood Transfusion Research Center, High Institute for Research and Education in Transfusion Medicine, Tehran, Iran

**Keywords:** Human plasma, Proteins, IgG, Coagulation factors, Fractionation

## Abstract

**Context:**

The major motivation for the preparation of the plasma derived biological medicine was the treatment of casualties from the Second World War. Due to the high expenses for preparation of plasma derived products, achievement of self-sufficiency in human plasma biotechnological industry is an important goal for developing countries.

**Evidence Acquisition:**

The complexity of the blood plasma was first revealed by the Nobel Prize laureate, Arne Tiselius and Theodor Svedberg, which resulted in the identification of thousands of plasma proteins. Among all these proteins, four of which are commercially important for production due to significant need of patients. These four products are: albumin, IgG, factor VIII, and Factor IX. The starting material for the production of biological drugs from plasma is natural which is different from synthetic starting material. So, the quality of plasma as starting material plays an important role in the quality of final product. Introducing new techniques for preparation of the biological drugs from human plasma has resulted in the improvements in purity of products, higher safety, and yield noticeably. Still, the backbone of the modern plasma fractionation technique is mainly based on cold ethanol fractionation of the human plasma that is almost the same as fractionation of crude oil, breaking it down into its components. The demand for IgG for treating immune deficiencies and coagulation factor VIII for hemophilia A determines how to design the plasma fractionation industry in terms of capacity. Nowadays, cold ethanol fractionation has followed by chromatographic methods, since they offer higher purity. In this review, we describe different methods of plasma fractionation such as cold ethanol fractionation, gel filtration, fractionation by salt, and fractionation by polyethylene glycol. There is no doubt that the four main products of human plasma are albumin, IgG, coagulation factor VIII, and IX, which their methods of separation from human plasma have been explained in this paper.

**Conclusions:**

It can be concluded that plasma fractionation with ethanol at low temperature for the preparation of the main human plasma biological components including albumin, IgG, coagulation factors VIII, and IX is still the most widely used method at an industrial scale. Nowadays, this method is being used in combination with different chromatographic techniques in order to achieve a higher quality and the yield.

## 1. Background

Nowadays, biological drugs medicines prepared from the human plasma play a vital role in the treatment of patients with different diseases (1-3). By the beginning of the new century, about 500,000 kg of human serum albumin (HAS) and 40,000 kg of intravenous immunoglobulin (IVIG) is being produced per year. For the production of such amounts of albumin and IVIG, more than 20 million liters of the starting material which is human plasma is needed. If we notice to more than $7 billion annual productions of these human plasma products, it can be concluded that implementation of new technology in the production of such biological drugs is a necessity (4,5) with respect to Good Manufacturing Practice (6). Also, virus inactivation should be carried out during the procedure of production (7-9).

Treatment of more than thousands casualties from world war II was a trigger for the preparation of biological drugs such as albumin and immunoglobulin from human plasma (10-12). For these reasons different studies on developing procedures for the preparation of “human plasma derived biological drugs” were carried out (13,14). One of the methods introduced for proteins’ separation from human plasma is salting out. For this purpose, ammonium sulfate as a salt can be added at different concentrations to the solution of human plasma for precipitation of the protein. The addition of ammonium sulfate should be repeated with different concentrations in order to obtain specific protein after each addition of the salt. Lactate of 2-ethoxy-6,9-diamino-acridine is another compound which can be used to decrease the solubility of proteins in human plasma, following to the addition of which insoluble protein can be separated by centrifugation. The other technique for plasma derived protein separation is fractionation of plasma by polyethylene glycol. In all techniques of plasma derived biological drugs preparation, virus inactivation is an essential step (15).

In our study, by this method, intermediate sources for preparation of fibrinogen, α1-antitrypsin, albumin, IgG, IgA, and IgM were achieved (16). Affinity and ion exchange chromatography are suitable for separation of labile protein such as coagulation factors. These techniques are suitable for the preparation of plasminogen and coagulation factors IX, VII, and immunoglobulin from human plasma (12,13,17-20). The main implementation of plasma derived biological drugs has been shown in (Table 1).

**Table 1:** Usage of plasma derived biological medicines

** Plasma Component**	**Clinical Use**
Albumin IgG Factor VIII Factor IX complex α_1_-anitrypsin Antithrombin III	Shock, Burns, Hypoalbumenia Loss of blood Passive prophylaxis Immune deficiency disorders Immune thrombocytopenic purpura Hemophilia A Hemophilia B Factor II and Factor X deficiencies Hereditary deficiencies Congenital deficiency

The most important biological medicines which can be prepared from human plasma are albumin,Immunoglobulin G, coagulation factor VIII, and coagulation factor IX. There are some other products which can be separated from blood but with less importance for patient’s treatment, due to fewer numbers of patients with specific deficiency of that compoundsuch as α1-antitrypsin, anti thrombin III, ceruloplasmin, and plasminogen (13,21,22). The amount needed for these compounds is much less than albumin and IgG. For this reason, a method with a higher capacity of preparation and possibility of implementation in an industrial scale is the most important factor.

Development of a process for separation of biological medicines such as albumin and IgG was first suggested by Edwin Cohn from Harvard University. His project was “plasma fractionation”, in order to prepare products such as albumin and IgG for the treatment of soldiers suffering from shock and burns, which made them needing for the blood volume expander. Nowadays, most of the modern human plasma fractionation industries are based on Cohn’s method. Selfsufficiency in plasma-derived biological medicines from national plasma has a high priority in developing countries (23-27).

## 2. Human Plasma Fractionation

The first step for human plasma protein separation is plasma fractionation. Cohn and his colleagues were the pioneer in plasma fractionation. They carried out plasma fractionation at low temperature by the addition of ethanol from 8% to 40% V/V at the end for separation of albumin (28). In Cohn’s method, five fractions could be obtained which are from fraction I to fraction V. The main component in each fraction has been shown in Table 2. Fraction I to V were prepared by adjusting parameters such as the concentration of ethanol, concentration of protein, temperature, and pH; concentration of ethanol from 8% reaches to 40% in preparation of fraction V. Nowadays, implementation of plasma fractionation is carried out by following chromatographic techniques (29).

**Table 2:** Percentage of the main proteins in different fractions archived by plasma fractionation

** No. of Fraction **	** % Protein **	** Components**
I II+III IV-1 IV-4 V	5-10 25 5-10 5-10 50-60	Fibrinogen Clotting factor VIII IgG IgA IgM α-and β-globulins α_1_-antitrypsin Antithrombin III Complement components Ceruloplasmin Haptoglobin Transferin Albumin

**Figure 1:**
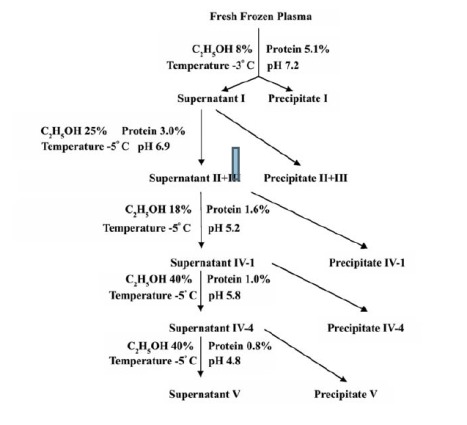
Processing of human plasma from fraction I to V

**Figure 2:**
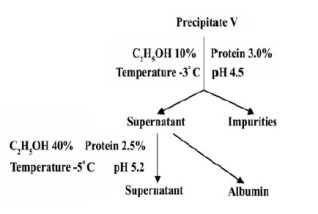
Rework process on Fraction V

For obtaining fraction I to V by cold ethanol fractionation method (Cohn’s method), adjustment of five parameters play an important role. These five parameters which have to be adjusted during plasma conditioning are pH, the concentration of ethanol, ionic strength, temperature, and concentration of protein (30). (Figure 1) shows the condition for adjustment of these key parameters for separation of fraction I to V from human plasma (28). In Figures 1 and 2, the percentage of ethanol is the concentration of ethanol in the final solution and the protein percent is the total protein percentage in the solution.

At the end of fractionation, a rework (*i.e.* a technical term in the fractionation of the plasma industry which doesn’t mean a work that was repeated) has to be done on fraction V to get rid of impurities such as lipoprotein to obtain albumin. The process for rework on fraction V is shown in Figure 2.

There are two reworks in plasma fractionation, one rework is done for fractions II+III and the other rework for fractions V+VI such that in the first rework fraction II can be separated and in the second rework, fraction V can be separated.

Fraction II+III is a good intermediate source for the preparation of immunoglobulin G (IgG) but only for getting rid of impurities further fractionation step which is rework II+III was carried out. The same is for fraction V+VI, which is a good source of albumin preparation and only for getting rid of tiny amounts of lipoproteins and glycoproteins the fractionation was carried out further.

## 3. Gel Filtration Plasma Fractionation

Among the methods which could be applied for the preparation of biological plasma derived drugs is gel filtration (31). Currently, this technique is being used by biochemical researchers for protein purification, when the size of protein matters. Gel filtration is a chromatographic method that is based on the differential steric interaction of the proteins with a gel medium due to differences in molecular size. Gel filtration technique can either be used for group separation which is the separation of molecules with a large difference in molecular size, or for fractionation of molecules with molecular size relatively close to each other termed fractionation of proteins. In fractionation of human plasma by gel filtration, those molecules with molecular sizes relatively close to each other can be separated. One of the advantages of this method is behaving gently with protein and causes no denaturing effects on proteins. The recovery of protein by this method is almost complete. Alimitation of this method for human plasma fractionation is its low flow rate as this would be a major limitation when a large amount of plasma has to be processed.

Due to above mentioned reason, gel filtration cannot be a suitable method when the separation of the main plasma proteins such as albumin and immunoglobulin is pursued. But, this method has been used for the purification of the anti haemophilic factor from cryoprecipitate.

## 4. Human Plasma Fractionation by polyethylene Glycol

Apart from the above mentioned procedures for human plasma fractionation such as using ammonium sulfate, Rivanol and caprylic acid precipitation, or fractionation by cold ethanol, or gel filtration method, using polyethylene glycol is another method for plasma fractionation. This polymeric non-toxic compound is soluble in water. The results of our study (16) in the fractionation of human plasma by polyethylene glycol are shown in Table 3.

**Table 3:** Distribution of the plasma proteins in PEG fractions (mg.dL^-1^)

** Proteins **	**Cryoprecipitate**	**5% PEG **	**10% PEG **	**20% PEG**	** Final Supernatant **
Total Protein Fibrinogen α1-Antitrypsin Albumin IgG IgA IgM	6050 181 162 3500 715 152 120	302(5.0%) 117(64.6%) 2(1.2%) 110(3.1%) 24(3.4%) 5(3.3%) 8(6.7%)	1210(20.0%) 31(17.1%) 5(3.1%) 115(3.3%) 585(81.8%) 45(29.6%0) 35(29.2%)	660(10.9%) 4(2.2%) 8(4.9%) 140(4.0%) 96(13.4%) 80(52.6%) 2(1.7%)	3510(58.0%) - 120(74.1%) 2980(85.1%) 5(0.7%) 26(17.1%) -

In our work fractionation of human plasma by different concentration of polyethylene glycol has been studied, and fractionation of human plasma was carried out by the addition of three different percentage of polyethylene glycol (5%, 10%, and 20%). It is noticeable from (Table 3) that in 5% polyethylene glycol, albumin is the main contaminant and fibrinogen is the main protein in this fraction. Distribution of α1-antitrypsin , fibrinogen, albumin, IgG, IgA, and IgM is shown in Table 3.

## 5. Plasma Fractionation by Ammonium Sulfate

There are not many salts which can be used as salting out method for human plasma fractionation. Actually, ammonium sulfate is one of the salts that are being used widely for protein separation. Separation of fibrinogen from human plasma can be carried out by repeated precipitation of plasma by ammonium sulfate. The concentration of am monium sulfate can be varied from 58% to 73% during precipitation, and it depends on the concentration of protein. Apart from using ammonium sulfate as precipitating agent, it could also be used in crystallization methods. For example, albumin is one of the human plasma proteins that can be crystallized by this technique.

The combination of precipitation by ammonium sulfate with other purification methods such as ion exchange chromatography or affinity chromatography for protein purification can be applied, as well.

 Ammonium sulfate along with Rivanol which is the lactate of 2-ethoxy-6,9-diamino a cridine is a beneficial in human plasma fractionation while using it in combination with octanoic acid has also has been reported.

## 6. Human Serum Albumin Preparation from Plasma

For albumin purification from human plasma, all types of plasma (outdated or fresh frozen plasma) can be used as starting material (32-34). Pasteurization at 60ºC for 10 hours is almost the only technique which is being used for virus inactivation of albumin. Different combinations of ion exchange chromatography have been used for albumin preparation such as DEAE-Sephadex A-50, DEAE-Sepharose CL-6B, or QAE Sephadex A-50 (35).

The best intermediate source for albumin preparation is the Cohn’s fraction V, otherwise by the concentration of 150 g.L^-1^ polyethylene glycol, most impurities of the human plasma such as globulins may be precipitated (16).

Albumin may be purified in one step by chromatography using QAE-Sephadex A-50 gel. The gel should be swollen in one molar sodium acetate. Also elution of albumin can be carried out by sodium acetate buffer. By this method the amount of polymer and aggregate of albumin is less than 5%. The prepared albumin due to virus inactivation should be pasteurized at 60ºC for 10 hours. For thermal resistance of the protein during pasteurization, an equal concentration of the sodium octanoate and sodium a cetyltryptophanate should be added to the protein solution. Using sodium a cetyltryptophanate and sodium octanoate as stabilizers for heat resistance of albumin during pasteurization is safe, and these two stabilizers are allowed to be used for albumin formulation based on United States Pharmacopeia (USP) and British Pharmacopeia (BP), as well.

## 7. Immunoglobulin G Preparation

Immunoglobulins may be separated from different sources of plasma (36-38).The best source for purification for human immunoglobulin G is the fraction II of the Cohn’s method (39).Virus inactivation is an important step during production procedure (40-42) and one of the methods for virus inactivation of IgG is acidic pH (43). Purification is also possible directly from human plasma treated with polyethylene glycol. For this purpose human plasma may be treated by different concentration of polyethylene glycol at neutral pH. Although we have tried a variation of polyethylene glycol concentrations from 5 to 20% , but the optimum concentration for separation of immunoglobulin G was found to be 10%, which most human globulins are precipitated at pH7 with this compound (16).

Precipitation of immunoglobulin was carried out by centrifugation at 1500 Xg for 20 min. Chromatography is the main technique for preparation of immune globulins. With a notice to the pI of immunoglobulin G which is 6.6-7.2, cation or anion exchange chromatography may be applied depending on the pH to be applied. At pH below pI protein has the positive charge, so cation exchange chromatography may be used, and at pH above pI protein has the negative charge, therefore anion exchange chromatography can be applied. Positively charged immunoglobulin G at pH below 6.6 can bind to the cation exchanger such as CM-Sephadex. At higher pH, the bound IgG can be eluted.

Negatively charged immunoglobulin G at pH above 7.2 can bind to the anion exchanger such as DEAE-Sephadex. So for purification of IgG both cation and anion exchange chromatography techniques can be carried out. Also, immunoglobulin may be produced by recombinant technique (44).

## 8. Coagulation Factor VIII Separation

There are numbers of coagulation factors involved in the coagulation cascade. Deficiency of these coagulation factors may be congenital or acquired. Defective hemostasis has been noticed due to lack of these factors. The most known deficiency of coagulation factors is related to factor VIII and factor IX, which cause respectively hemophilia A, and hemophilia B disease.

Hemophilia A disease is due to deficiency or absence of factor VIII clotting activity. Attempts to purify FVIII by isoelectric precipitation were promising. Also, salting out procedures for preparation of factor VIII was successful. However, factor VIII preparation is mostly based on human plasma fractionation. The quality of plasma has a great impact on the product (45). The first step of plasma fractionation produces Cohn Fraction I, which the cryoprecipitate from this fraction is a suitable source as starting material for factor VIII production (46).

Basically, for further purification of factor VIII, chromatography and particularly affinity chromatography techniques are often used in order to isolate coagulation factor VIII (47,48). Ion exchange chromatography of factor VIII on DEAE-Fractogel (a co-polymer) produces a highly purified factor VIII. Researchers are looking for the improved methods that will increase the yield. These techniques include affinity chromatography as well as the use of monoclonal antibodies to bind procoagulant of factor VIII. Virus inactivation should be applied in factor VIII production procedure. In the procedure for the production of factor VIII different methods of heat treatment also in addition to solvent/detergent treatment are required. Virus inactivation method composed of solvent/detergent inactivates the lipid enveloped viruses (49). In this method, the solvent is tri-n-butyl phosphate and the detergent may vary, such as Tween 80, Triton X100, or sodium cholate.

## 9. Preparation of Coagulation Factor IX

For treatment of hemophilia B, pure factor IX or concentrated factor IX (*i.e.* prothrombin complex) may be used. Factor IX concentrate or prothrombin complex contains factor II, factor VII, factor IX, and factor X. All these factors are synthesized by the liver and the synthesis is vitamin K dependent.

Isolation of pure factor IX can be difficult somehow due to the physico-chemical characteristics ,the similarity of these four factors such as is oelectric point, molecular weight, and electrophoretic mobility (50).

For preparation of prothrombin complex, the supernatant of plasma after cryoprecipitation or supernatant of Cohn’s Fraction I may be used as the starting material.

Ion exchange chromatography seems to be suitable for the preparation of prothrombin complex by an anion exchanger such as DEAE-Sephadex A-50. Different ratios of the gel per liter of plasma supernatant have been tried and the optimal adsorption of these four coagulation factors has been achieved when one to two gram of the gel has been used per liter of supernatant. Washing and elution have been carried out by a solution of sodium chloride in citrate buffer. After adsorption of proteins to the gel, it was washed with 0.2 M sodium chloride in 0.01 M citrate at neutral pH. In this way unwanted proteins can be washed away from the DEAE-Sephadex A-50, but not the desired factors II, VII, IX, and X. For elution of the four coagulation factors 0.2 M sodium chloride in 0.015 citrate buffer at neutral pH was used. In order to regenerate the column, increasing the molarity of the sodium chloride from 0.2 M to 2.0 M causes the elution of unwanted protein due to the increasing the ionic strength.

Desalting of prothrombin complex after ion exchange chromatography may be carried out by gel filtration on Sephadex G-25. Formulation of the prothrombin complex after desalting is the next step. Adjustment of conductivity may be carried out by sodium chloride solution and the pH should be adjusted to 7.0. It should be noticed that during prothrombin complex preparation procedure, virus inactivation has to be implemented (51) and the product should be sterile filtered.

In order to prepare pure coagulation factor IX, Cohn’s Fraction I or prothrombin complex may be used. Anion exchange chromatography for purification of factor IX from prothrombin complex by DEAE Sepharose CL-6B gel is possible. In addition, affinity chromatography using Heparin-Sepharose was found to increase the specific activity of the factor IX noticeably (52).

## 10. Conclusions

In preparation of the proteins from human plasma by fractionation, apart from major issues such as safety of products and effective therapeutic value, one of the main concerns is the cost effectiveness of the procedure due to the cost of human plasma as a starting material. Noticing to this matter, investigations on plasma protein fractionation is oriented to separate as many proteins as possible in order to neutralize the cost of plasma.

It can be concluded that plasma fractionation with ethanol at low temperature for the preparation of the main human plasma biological components including albumin, IgG, coagulation factors VIII, and IX is still the most widely used method at an industrial scale. Nowadays, this method is being used in combination with different chromatographic techniques in order to achieve a higher quality and the yield.

## Acknowledgements

The authors are thankful to the Blood Transfusion Research Center, High Institute for Research and Education in Transfusion Medicine for their support.

## Authors’ contributions

Kamran Mousavi Hosseini conceived, Kamran Mousavi Hosseini and Mehran Ghasemzadeh collected and Kamran Mousavi Hosseini drafted the manuscript. Kamran Mousavi Hosseini and Mehran Ghasemzadeh analyzed the data. Both authors have read and approved the final manuscript.

## Funding/Support

Financial support for this work was provided by Blood Transfusion Research Center, High Institute for Research and Education in Transfusion Medicine.
